# Structural Evolution of Primate Glutamate Dehydrogenase 2 as Revealed by In Silico Predictions and Experimentally Determined Structures

**DOI:** 10.3390/biom14010022

**Published:** 2023-12-23

**Authors:** Ionela Litso, Andreas Plaitakis, Vasiliki E. Fadouloglou, Mary Providaki, Michael Kokkinidis, Ioannis Zaganas

**Affiliations:** 1 Neurology/Neurogenetics Laboratory, School of Medicine, University of Crete, Voutes, 71003 Heraklion, Greece; medp2012038@med.uoc.gr (I.L.); andreasplaitakis@gmail.com (A.P.); 2 Department of Molecular Biology and Genetics, Faculty of Health Sciences, Democritus University of Thrace, 68100 Alexandroupolis, Greece; fadoulog@mbg.duth.gr; 3 Institute of Molecular Biology and Biotechnology, Foundation of Research and Technology-Hellas, 70013 Heraklion, Greece; providak@imbb.forth.gr (M.P.); kokkinid@imbb.forth.gr (M.K.); 4 Department of Biology, University of Crete, Vasilika Vouton, 71409 Heraklion, Greece

**Keywords:** glutamate dehydrogenase, AlphaFold, protein structure prediction, primate evolution

## Abstract

Glutamate dehydrogenase (GDH) interconverts glutamate to a-ketoglutarate and ammonia, interconnecting amino acid and carbohydrate metabolism. In humans, two functional GDH genes, *GLUD1* and *GLUD2*, encode for hGDH1 and hGDH2, respectively. *GLUD2* evolved from retrotransposition of the *GLUD1* gene in the common ancestor of modern apes. These two isoenzymes are involved in the pathophysiology of human metabolic, neoplastic, and neurodegenerative disorders. The 3D structures of hGDH1 and hGDH2 have been experimentally determined; however, no information is available about the path of GDH2 structure changes during primate evolution. Here, we compare the structures predicted by the AlphaFold Colab method for the GDH2 enzyme of modern apes and their extinct primate ancestors. Also, we analyze the individual effect of amino acid substitutions emerging during primate evolution. Our most important finding is that the predicted structure of GDH2 in the common ancestor of apes was the steppingstone for the structural evolution of primate GDH2s. Two changes with a strong functional impact occurring at the first evolutionary step, Arg443Ser and Gly456Ala, had a destabilizing and stabilizing effect, respectively, making this step the most important one. Subsequently, GDH2 underwent additional modifications that fine-tuned its enzymatic properties to adapt to the functional needs of modern-day primate tissues.

## 1. Introduction

Glutamate dehydrogenase (GDH) reversibly interconverts glutamate to a-ketoglutarate and ammonia using NAD(P)+ as cofactors [1,2]. The enzyme interconnects carbon and nitrogen metabolism and is found in almost all living organisms [3,4,5]. In eukaryotes, GDH is abundantly expressed in mitochondrial matrix, where it is involved in glutamate metabolism linking it with energy homeostasis [6,7,8,9]. Specifically, α-ketoglutarate, produced via oxidative deamination of glutamate, feeds the Krebs Cycle, serving anaplerotic functions and leading to ATP synthesis [3].

In addition to the *GLUD1* gene (encoding for hGDH1), humans possess *GLUD2* (encoding for hGDH2), an intronless X-linked gene thought to have evolved through retrotransposition of a spliced *GLUD1* mRNA [10]. Subsequent phylogenetic studies revealed that the retrotransposition of the *GLUD1* gene to the X chromosome occurred during primate evolution more than 23 million years ago [11]. After emerging in the common ancestor of humans and other modern apes, *GLUD2* underwent rapid evolutionary adaptation concurrently with brain evolution [11,12,13,14]. This adaptation involved 15 amino acid substitutions in the mature hGDH2 that provided unique functional properties [15].

Both *GLUD* genes encode for a 558 amino acid-long polypeptide sequence. The first 53 amino acids located on the N-terminus domain correspond to the leader peptide, which is responsible for the transportation of the enzyme inside the mitochondrial matrix [16]. The mature hGDH1 and hGDH2 isoenzymes, resulting from cleavage of the leader peptide inside the mitochondrion, share all but 15 of their 505 amino acids [10]. Despite its sequence similarity to hGDH1, hGDH2 has unique enzymatic and regulatory properties, including GTP resistance, relatively low basal activity markedly responsive to activation by ADP and/or L-leucine, lower optimal pH, and relative sensitivity to thermal inactivation [15]. As shown by enzymatic studies, these highly divergent properties are to a large extent related to Arg443Ala and Gly456Ala, 2 of the 15 amino acid substitutions that occurred during hGDH2 evolution [17,18,19].

In addition to distinct enzymatic and regulatory properties, hGDH2 displays a unique expression pattern. hGDH1 is encoded by the housekeeping *GLUD1* gene and is expressed in all human tissues, with the highest levels found in the liver. Gain-of-function amino acid changes lead to the hyperinsulinism hyperammonemia syndrome [20,21,22,23], a serious metabolic disorder with childhood onset. On the other hand, hGDH2 is expressed mainly in the human brain, kidney, testes, and steroidogenic organs while showing low expression levels in the human liver [24]. Recently, the possibility has emerged that hGDH2 is involved in the pathogenesis of neurodegenerative and neoplastic disorders [25,26].

**Figure 1:**
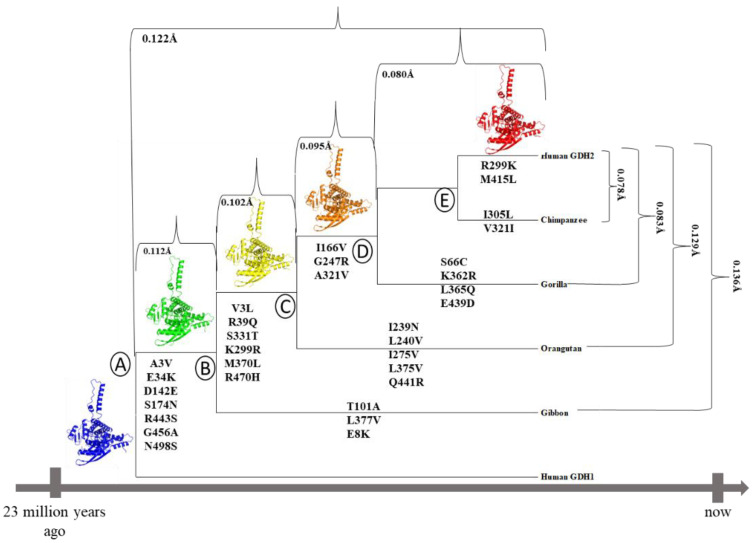
Phylogenetic tree, based on the *GLUD2* sequences encoding the mature polypeptide, constructed by the Molecular Evolutionary Genetics Analysis program [42] using the neighbor-joining method. On its branches, the amino acid substitutions that led to the current GDH2 proteins in great apes are depicted. Numbers refer to the RMSD values for each comparison. Cartoon diagrams were created using the PyMOL software (The PyMOL Molecular Graphics System, Version 2.5, Schrödinger, LLC, New York, NY, USA).

The 3D structure of hGDH1 [27] and the structures of several other mammalian and non-mammalian GDH1s [28,29,30,31,32,33,34,35,36,37] have been determined by X-ray crystallography. The mammalian GDH1 structure is a symmetric homo-hexamer, with each subunit consisting of the N-terminal glutamate-binding domain, the NAD+-binding domain, the antenna, the pivot helix, and the C-terminal helices [38]. Recently, we have determined the crystal structure of the hGDH2 protein at 2.9 Å resolution, showcasing important differences compared to hGDH1 [39]. However, no information is available about the structure of GDH2 in modern apes, other than humans, or in their, now extinct, common ancestors (nodes B, C, D, and E in Figure 1). Importantly, the modern hGDH1 corresponds to the original GDH sequence present 23 million years ago, from which the line that led to the modern primate GDH2 emerged. This evolutionary conservation shows that GDH1 is a crucial metabolic enzyme with little tolerance for changes.

Advanced protein structure prediction algorithms have been recently developed to supplant experimentally determined protein structures. One such algorithm is AphaFold, developed by DeepMind, which uses artificial intelligence to accurately predict protein structures from their amino acid sequence [40,41]. Here, we examine the accuracy of the predicted hGDH1 and hGDH2 AphaFold Colab models by comparing them with the experimentally determined human enzyme structures. Furthermore, AlphaFold Colab is used to predict the structures of GDH2 of modern apes and their ancestors, going back 23 million evolutionary years. Finally, we present the effects of the amino acid substitutions that occurred at each evolutionary step.

## 2. Materials and Methods

### 2.1. Phylogenetic Tree Analysis

The phylogenetic tree, based on the *GLUD2* sequences encoding the mature polypeptide, was constructed by the Molecular Evolutionary Genetics Analysis (MEGA) program [42] using the neighbor-joining method (Figure 1). On the branches of this tree, the amino acid substitutions that led to the emergence of current GDH2 proteins in great apes are depicted.

### 2.2. Protein Structural Prediction and Analysis

The experimental crystallographic structure of hGDH1 and hGDH2 was retrieved from the Protein Data Bank (pdb code “1L1F” and “6G2U”, respectively).

These structures were determined from crystals grown under the following conditions: (1) hGDH1: 1% (*w/v*) octyl-b-glucopyranoside, 1 mM sodium azide, 50 mM sodium chloride, 8% (*v/v*) methyl pentanediol, 0.1 M sodium phosphate (pH 7.3), and 0.1 M sodium phosphate buffer (pH 7.0); (2) hGDH2: 8% PEG 8000, 15% MPD, 0.4 M NaCl, and 0.1 M phosphate buffer (pH 7.0) [27,39].

AlphaFold, accessed on 29 January 2022, (https://colab.research.google.com/github/sokrypton/ColabFold/blob/main/AlphaFold2.ipynb) was used to predict the structures of GDH2 proteins in modern-day great apes and in extinct primates. This server predicts protein structure, including alpha helices and beta sheets, from their amino acid sequence, using a simplified version of AlphaFold v2.0 that does not require homologous structures (templates).

The best five models were selected according to the ranking by the predicted local-distance difference test (pLDDT) confidence values (higher = better to lower = worse). The AlphaFold pLDDT scores for the proteins studied are shown in Table 1. The resulting models were examined, aligned, and compared to each other, and to the experimentally determined structures using PyMOL. The command “super” was used comparing protein backbones. To evaluate the differences between predicted or experimentally determined structures, we used the root-mean-square deviation (RMSD) values resulting from the alignments. An RMSD value below <1.8 Å was considered as suggestive of high accuracy.

### 2.3. Mutational Analysis

Mutant GDH2 structural stability for each evolutionary step was estimated based on changes in free energies, ΔΔG (kcal/mol). The predicted structures of mutant GDH2s generated from AphaFold were used to calculate ΔΔG changes. Five different webservers were used: the sequence-based iSTABLE (http://predictor.nchu.edu.tw/iStable/about.php) [43], the structure-based PremPS (https://lilab.jysw.suda.edu.cn/research/PremPS/) [44], MaestroWEB (https://pbwww.services.came.sbg.ac.at/maestro/web/) [45], SDM (http://marid.bioc.cam.ac.uk/sdm2) [46], and DynaMut (http://biosig.unimelb.edu.au/dynamut/) [47]. All webservers were accessed on 4 March 2022.

The evaluation of the structural stability of various GDH2s was based on an analysis of the results of the five methodologies when they reached a majority consensus. In the framework of these methodologies, the application of iSTABLE, PremPS, MaetroWEB, SDM, and DynaMut provided the estimate of the unfolding and total free energy as well as the vibrational entropy (Table 2). Differences in the results obtained by these servers when calculating the stabilizing/destabilizing impact of each amino acid change are due to the use of different algorithms.

iSTABLE, which uses a support vector machine (SBVM) system, combines the results from different predictors such as iMUTANT and MUpro to determine the effect of point mutations on protein stability [43], by calculating the difference in folding energy change (ΔΔG in Kcal/mol) between the wild-type and the mutant protein. DynaMut predicts changes in protein stability (ΔΔG in Kcal/mol), variation in entropy energy, and changes in protein flexibility, and allows visualization of non-covalent molecular interactions [47]. The webserver uses Bio3D [48] and ENCoM [49] approaches to perform a Normal Mode Analysis (NMA), providing rapid and simplified access to analyses about protein motions. ΔΔG > 0 corresponds to a stabilizing effect, whereas ΔΔG < 0 corresponds to a destabilizing effect. PremPS predicts changes in protein stability (ΔΔG in Kcal/mol) as well as the location of the mutation, either in the hydrophobic core or on the protein surface [44]. The ΔΔG prediction is based on a random forest regression method that uses evolutionary and structure features. Positive ΔΔG values indicate a destabilizing effect on protein stability, whereas negative ΔΔG values indicate a stabilizing effect. The Multi AgEnt STability pRedictiOn (MAESTRO) webserver estimates the changes in unfolding free energy upon point mutation through a machine learning system [45]. ΔΔG > 0 corresponds to a destabilizing effect, whereas ΔΔG < 0 corresponds to a stabilizing effect. Site Directed Mutator (SDM) uses environment-specific amino acid substitution frequencies within homologous protein families to calculate a stability score, which is analogous to the free energy difference between the wild-type and mutant protein [46]. Positive ΔΔG values indicate a stabilizing effect, whereas negative ΔΔG values indicate a destabilizing effect.

### 2.4. Sequence Alignment

**Figure 2:**
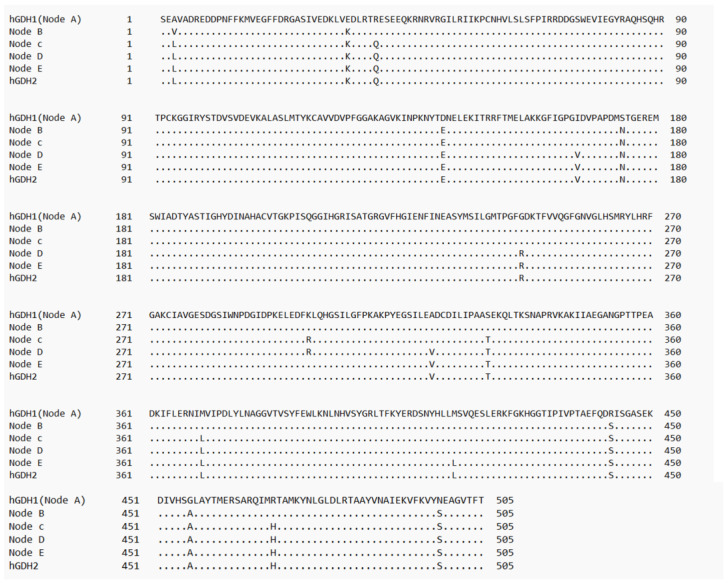
Schematic representation of multiple sequence alignment of glutamate dehydrogenase protein sequences during evolution. The dots indicate amino acid identities among the enzymes. Nodes A to E correspond to nodes A to E in Figure 1. This diagram was created using the BLASTP tool.

The five amino acid sequences corresponding to the evolutionary steps, along with hGDH1 and hGDH2, were aligned using the BLASTP tool (https://blast.ncbi.nlm.nih.gov/Blast.cgi?PAGE=Proteins), accessed on 21 November 2023. (Figure 2).

## 3. Results

### 3.1. AlphaFold Predictions vs. Experimentally Determined hGDH1 and hGDH2 Structures

**Figure 3:**
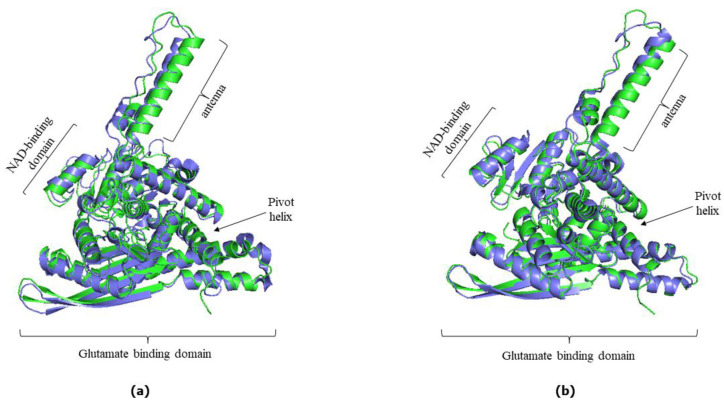
(**a**) Superimposed structures of experimentally determined hGDH1 (green, PDB code: 1L1F) and hGDH1 AlphaFold Colab-derived structure model (blue). The RMSD value between the two superimposed structures was estimated to be 1.745 Å. (**b**) Superimposed structure of experimentally determined hGDH2 (green, PDB code: 6G2U) and hGDH2 AlphaFold Colab structure model (blue). The RMSD value between the two superimposed structures was estimated to be 0.895 Å. In both (**a**,**b**), the individual domains found in each subunit of the hexameric enzyme are highlighted. The PyMOL Molecular Graphics System, Version 2.5, Schrödinger, LLC, was used to create cartoon models.

AlphaFold provided a satisfactory prediction of the experimental 3D structures of the hGDH1 and hGDH2 protein (Figure 3), as the predicted protein structures showed all the important domains found in each subunit of the hexameric glutamate dehydrogenases. These domains include a glutamate binding region towards the N terminus, a NAD binding domain, and a regulatory domain consisting of the antenna and the pivot helix (Figure 1).

We initially explored whether the hGDH1 and hGDH2 structures predicted from their sequences using AlphaFold Colab were accurate. To answer this, the predicted hGDH1 structure derived from AlphaFold Colab and the experimentally determined hGDH1 structure (PDB entry 1L1F) were superimposed (at a total of 3418 atoms) using the PyMOL “super” command. The RMSD value between the two superimposed structures was estimated to be 1.745 Å (Figure 3a). Similarly, the predicted hGDH2 structure derived from AlphaFold Colab and the experimental hGDH2 structure (PDB entry 6G2U) were superimposed using PyMOL, at a total of 3278 atoms. The RMSD between the AlphaFold Colab structure and the experimental template was 0.895 Å (Figure 3b). Thus, the comparisons of the AlphaFold predicted structures with the experimentally determined structures highlight the ability of this approach to adequately predict the structures of the individual domains.

**Figure 4:**
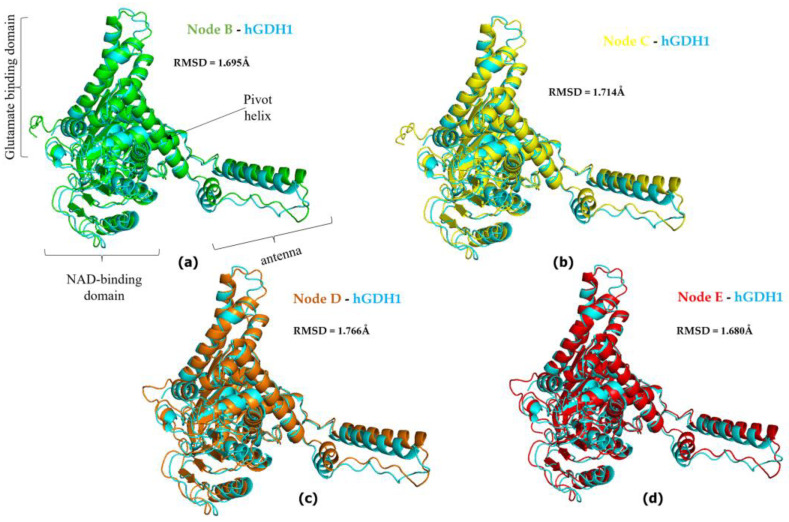
Superimposed hGDH2 AlphaFold Colab predicted structures during primate evolution to experimentally determined hGDH1. (**a**) Node B (green)—hGDH1(blue). (**b**) Node C (yellow)—hGDH1(blue). (**c**) Node D (orange)—hGDH1(blue). (**d**) Node E (red)—hGDH1(blue). The RMSD values are shown in the figure, next to each comparison. In (**a**), the individual domains found in each subunit of the hexameric enzyme are named. The PyMOL Molecular Graphics System, Version 2.5, Schrödinger, LLC, was used to create the cartoon models. Nodes A to E correspond to nodes A to E in Figure 1.

**Figure 5:**
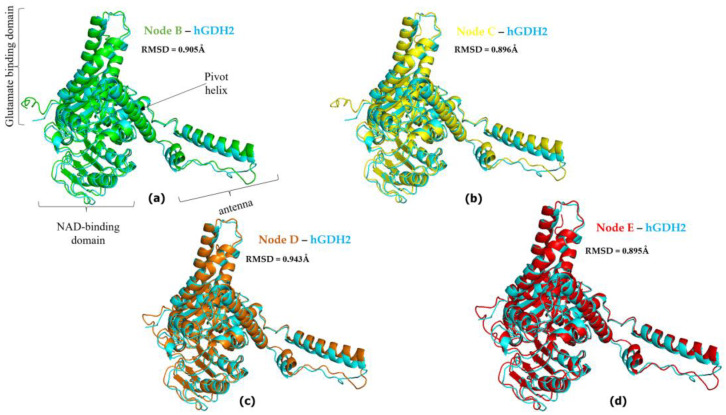
Superimposed hGDH2 AlphaFold Colab predicted structures during primate evolution to experimentally determined hGDH2. (**a**) Node B (green)—hGDH2 (blue). (**b**) Node C (yellow)—hGDH2 (blue). (**c**) Node D (orange)—hGDH2 (blue). (**d**) Node E (red)—hGDH2 (blue). The RMSD values are shown next to each comparison. In (**a**), the individual domains found in each subunit of the hexameric enzyme are named. The PyMOL Molecular Graphics System, Version 2.5, Schrödinger, LLC, was used to create the cartoon models. Nodes A to E correspond to nodes A to E in Figure 1.

Also, comparison of the AlphaFold-derived structures corresponding to nodes B, C, D, and E in Figure 1 with the experimentally determined hGDH1 and hGDH2 structures gave results comparable with those described above (Figure 4 and Figure 5). Specifically, the comparison of experimental hGDH1 with proteins predicted for nodes B, C, D, and E gave RMSD values of 1.695 Å, 1.714 Å, 1.766 Å, and 1.680 Å, respectively (Figure 4). For hGDH2, these values were calculated to be 0.905 Å, 0.896 Å, 0.943 Å, and 0.895 Å, respectively (Figure 5).

### 3.2. hGDH2 AlphaFold Colab Predicted Structures during Evolution That Led to Humans

The human *GLUD1* gene that encodes for hGDH1 has remained unchanged for the last 23 million years. This indicates that it is an ortholog of and essentially identical to the original GDH gene (node A, Figure 1) in the common ancestor of modern apes. Thus, we have good reason to support that the experimentally determined hGDH1 structure corresponds to that of the common ancestral enzyme.

In the common ancestor of humans and modern apes, seven amino acid substitutions occurred during the first evolutionary step following the retrotransposition event (node B, Figure 1). These were Ala3Val, Glu34Lys, Asp142Glu, Ser174Asn, Arg443Ser, Gly456Ala, and Asn498Ser. During the second evolutionary step, after the separation of the gibbon branch, six amino acid substitutions (Val3Leu, Arg39Gln, Lys299Arg, Ser331Thr, Met370Leu, Arg470His) appeared (node C, Figure 1). Finally, on the last two steps (nodes D and E, Figure 1), three (Ile166Val, Gly247Arg, Ala321Val) and two (Arg299Lys, Met415Leu) substitutions, respectively, led to the current hGDH2 protein in humans.

The GDH predicted structures corresponding to node A and node B were superimposed (at a total of 3171 atoms) using PyMOL and the RMSD value between the two models was 0.112 Å (Figure 1 and Figure S1). Similarly, the GDH2 node B and node C predicted structures were superimposed (at 3212 atoms) and the RMSD value was 0.102 Å (Figure 1 and Figure S1). The RMSD value between the superimposed GDH2 node C and node D structures (at 3088 atoms), as well as the GDH2 D and node E structures (at 31,997 atoms), were 0.095 Å and 0.080 Å, respectively (Figure 1 and Figure S1). Finally, GDH2 node A and node E predicted structures were superimposed (at 3088 atoms) and the RMSD value was 0.122 Å (Figure 1 and Figure S1).

### 3.3. Ape GDH2 AlphaFold Colab Predicted Structures and Comparison with Predicted hGDH2

**Figure 6:**
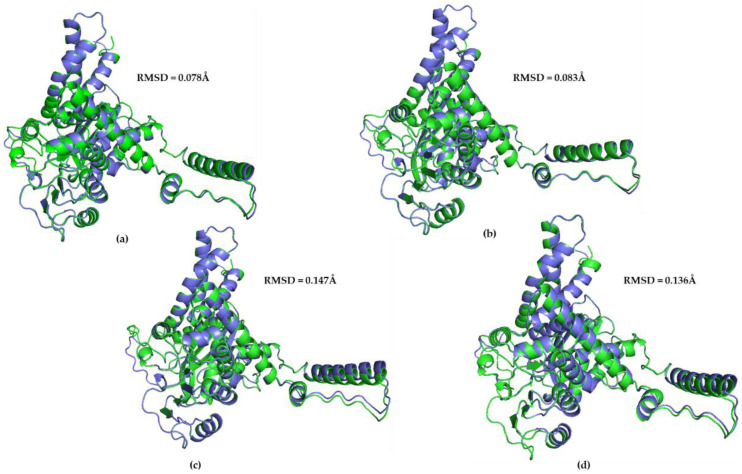
Superposition of the model structure corresponding to hGHD2 (blue) with every predicted structure model for each ape (green). (**a**) Chimpanzee—hGHD2. The RMSD value was estimated to be 0.078 Å. (**b**) Gorilla—hGHD2, RMSD value: 0.083 Å. (**c**) Orangutan—hGHD2, RMSD value: 0.147 Å. (**d**) Gibbon—hGHD2, RMSD value: 0.136 Å.

The predicted structure models for each ape (chimpanzee, gorilla, gibbon, orangutan) and the predicted structure model for hGHD2 were superimposed using PyMOL. The RMSD value between the chimpanzee predicted structure and the hGDH2 predicted structure was 0.078 Å (3100 atoms), whereas the RMSD value between the gorilla predicted structure and the hGDH2 predicted structure was 0.083 Å (3190 atoms). Correspondingly, the RMSD values from the superimposition of the gibbon predicted structures and the orangutan predicted structures with the hGDH2 predicted structure were 0.136 Å (3078 atoms) and 0.129 Å (3155 atoms), respectively (Figure 1 and Figure 6).

### 3.4. Ape GDH2 AlphaFold Colab Predicted Structures during Evolution

During apes’ evolution, after the separation of the gibbon branch (node B, Figure 1), three substitutions (Thr101Ala, Leu377Val, Glu8Lys) emerged and led to the establishment of the current gibbon GDH2 protein. Similarly, five amino acid substitutions (Ile239Asn, Leu240Val, Ile275Val, Leu375Val, Gln441Arg) appeared after the separation of the orangutan branch (node C, Figure 1) that led to the emergence of the current orangutan GDH2 enzyme. The establishment of the gorilla and chimpanzee protein was due to four (Ser66Cys, Lys362Arg, Leu365Gln, Glu439Asp) and two (Ile305Leu, Val321Ile) amino acid substitutions, respectively, after the separation of their phylogenetic branches (nodes D and E, respectively, Figure 1).

The model structure corresponding to the common ape ancestor was superimposed, using PyMOL, with every predicted structure model for each ape (Figure S2). The RMSD value between the chimpanzee protein and the common ancestor protein was 0.151 Å (3106 atoms), while the RMSD value between the gorilla predicted structure and the ancestor predicted structure was 0.139 Å (3180 atoms). Similarly, RMSD values from the superposition of the gibbon protein and the orangutan protein with the common ancestor predicted structure were 0.109 Å (3289 atoms) and 0.147 Å (3280 atoms), respectively. These results are comparable to the same calculations for modern-day hGDH2 (0.122 Å).

### 3.5. Mutational and Intramolecular Interactions Analysis

**Figure 7:**
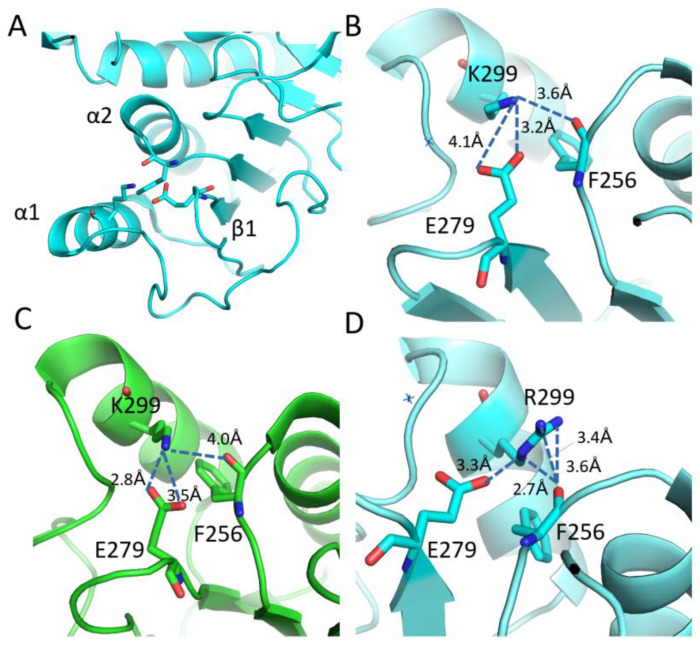
Effect of Lysine 299 substitution by Arginine. (**A**) The domain where residue 299 is located in the structure and secondary structure elements interconnected through interactions with residue 299. (**B**,**C**) Intramolecular interactions from position 299 (green GDH1/PDB ID 1L1F and cyan GDH2/PDB ID6G2U) when it is occupied by a lysine and (**D**) an Arginine residue (modelled in 6G2U PDB file), respectively. Lys 299 lies on α1 helix and makes H-bonds with the carbonyl group of Phe 256 (α2 helix) and electrostatic interactions/H-bonds with Glu279 (β1 strand). When this position is occupied by an Arginine, the number of possible interactions with the same elements increases.

In total, 18 evolutionary amino acid substitutions present in the protein surface (with 15 of them still present in modern humans) were analyzed to predict the result of each amino acid substitution during hGDH2 evolution (nodes A to E, Figure 1). The evaluation of the effect of the amino acid substitutions on protein stability by consensus indicated that 50% of the mutated sites generated a stabilizing effect and 50% generated a destabilizing effect (Table 2 and Table S1, Figure 7 and Figure S3). Since our findings revealed that the amino acid substitutions occurring during great apes’ evolution were altering the free energy and the dynamics of the enzyme, we aimed to investigate the impact of these amino acid replacements on the intramolecular interactions (Table 3). Structure-based analysis by DynaMut, using the hGDH1 structure as a template, revealed that the amino acid substitutions were significantly affecting these intramolecular interactions (Table 3).

Specifically, during the separation of the phylogenetic branches of the Old World apes and the African green monkey, seven amino acid changes emerged (node A, Figure 1). Ala3Val, Asp142Glu, Ser174Asn, and Gly456Ala increased protein stability based on the consensus of methods (Table 2, Figure S4). On the other hand, Glu34Lys, Arg443Ser, and Asn498Ser decreased protein stability (Table 2, Figure S4). These seven amino acid changes led to the loss and gain of bonds and interatomic interactions, as shown in Table 3.

Five of the six amino acid substitutions that occurred after the separation of the gibbon phylogenetic branch (node B, Figure 1, Table 2) had a destabilizing effect on protein structure (Val3Leu, Arg39Gln, Lys299Arg, Ser331Thr, Arg470His), with only Met370Leu increasing protein stability. Bonds and interactions lost and gained by these amino acid changes are shown in Table 3, with the Arg39Gln change producing no gain or loss of intramolecular interactions. Specifically, for the Lys299Arg change, Lysine 299 from α1 helix can make H-bonds and electrostatic interactions with residues from α2 helix and β1 strand (Figure 7), therefore connecting all these elements together. The Lys299Arg substitution leads to even more interactions (Figure 7D) and a higher intraconnection of these secondary structure elements (Figure 7).

During the separation of the orangutan phylogenetic branch (node C, Figure 1), three amino acid substitutions emerged. Ile166Val and Gly247Arg were found to destabilize the protein structure, whereas Ala321Val was found to have an opposite stabilizing effect (Table 2, Figures S4 and S5). These seven amino acid changes led to the loss and gain of bonds and interatomic interactions, As shown in Table 3, these three amino acid changes led to multiple changes in interatomic interactions.

Arg299Lys (reverting to the original amino acid) and Met415Leu, which emerged during the separation of the Homo branch from the chimpanzee branch (node E, Figure 1), decreased and increased protein stability, respectively (Table 2), and led to a loss and gain of interatomic interactions (Table 3).

## 4. Discussion

*GLUD2* is a novel gene that emerged though duplication in the hominoid ancestor approximately 23 million years ago [11] and underwent rapid evolutionary adaptation concurrently with primate brain evolution. The encoded human GDH2 (hGDH2) diverged substantially from its conserved hGDH1 ancestor, in its functional, expressional, and structural profile [17,18,19,50].

Although the 3D structures of modern hGDH1 and hGDH2 have been experimentally determined using X-ray crystallography [27,39], the detailed structural and functional properties of ancestral GDH2 enzymes that appeared during evolution are unknown. In this respect, we do not know if the primate GDH2 enzyme acquired its modern-day structural and functional characteristics upon its emergence more than 23 million years ago or during subsequent evolutionary steps. In addition, due to the lack of other experimental structures, it is presently unclear whether hGDH2 differs from that of other modern primates.

To approach this question in terms of 3D molecular structures, we used the AlphaFold server to obtain structure predictions based on amino acid sequence information. It is widely accepted that AlphaFold predictions are generally accurate and often comparable to the experimentally determined structures, even though this is not always the case [40,51]. The AlphaFold algorithm uses Al-ML to predict 3D model structures across 21 proteomes of human (98.5% of the human proteins) and non-human organisms [51,52]. However, it has not been widely used for the delineation of structures of proteins of extinct species, as is the case here. Initially, we used the amino acid sequence of hGDH1 and hGDH2 as templates to obtain the AlphaFold predicted structures of these proteins and to compare them with the experimentally determined hGDH1 and hGDH2 structures. These comparisons revealed that the AlphaFold predictions were fairly accurate, thus proving the ability of this approach to adequately predict the structures of the individual domains.

Then, to gain insight into the evolutionary emergence of hGDH2, we compared the AlphaFold predicted structures of GDH2 of modern-time apes and their now extinct ancestor. Our most important result using AlphaFold was that the predicted structure of GDH2 of the common ancestor of humans and other extant apes (chimpanzee, gorilla, orangutan, and gibbon) was the steppingstone for the structural and functional evolution of GDH2s in primates, with the first evolutionary step being associated with a higher RMSD value than subsequent steps. Indeed, judging by the RMSD values (Figure 1), the first evolutionary step was the most crucial one for the evolution of GDH2 in primates. In addition, we found that the GDH2 structure of the gibbon was more divergent from hGDH2 than those of great apes closely related to humans.

It is common in protein evolution that the most important properties are acquired upon emergence, otherwise, they become non-functional pseudogenes [53]. This initial evolutionary step before the separation of the human and gibbon lineages lasted approximately 5 million years and coincided with increasing functional properties of the primate brain [11]. Similar evolutionary processes to those of hGDH2 were in action for several other proteins that evolved during this period, such as opsins [54].

Also, given the differences in GDH2 structure between non-human primates and humans, this evolutionary step could reflect the diversification of brain function between primates. Humans are very close to chimpanzees at the genetic level (nucleotide difference of 1–2% at the level of the genome), making chimpanzees our closest living relatives [55,56]. However, despite the great nucleotide similarity, only 20% of the proteins are identical between the two species, even though research on this is ongoing [55]. An example of a protein that differs between humans and other apes is the digestive enzyme amylase. All vertebrates, including primates, express the enzyme in their pancreas. However, Old World monkeys and humans, but not New World monkeys, express a-amylase additionally in their saliva. The ability to express a-amylase in saliva in Old World monkeys, apes, and humans evolved after several duplications of the pancreatic amylase gene *AMY2* within the primate lineage [57].

In addition, we examined separately the effect of the amino acid substitutions emerging during apes evolution, using five different webservers, in accordance with other proteins, including the spike glycoprotein of SARS-CoV-2 [58]. Of note, we cannot make comparisons between different amino acid substitutions since results vary across different stability prediction webservers. In addition, there is no simple relation between ΔΔG and protein stability. Vlassi et al. [59] showed that protein cores can adapt to single amino acid substitutions without significant loss of stability as long as the volume change per substitution is limited to one methyl group. Also, they showed that during an evolutionary event, only single amino substitutions of this size causing minimum structural and functional destabilization are evolutionary acceptable. [59]

It is worth mentioning that two important evolutionary changes, Arg443Ser and Gly456Ala, had a destabilizing and stabilizing effect, respectively. These findings corroborate previous enzymatic studies by us and others that Arg443Ser and Gly456Ala, which occurred in this first step, before the separation of the gibbon lineage, gave GDH2 its most important functional properties [17,19,60,61]. These functional properties are low basal activity markedly activated by ADP and resistance to GTP inhibition, respectively [17,18,62]. There is evidence that these regulatory properties provided a novel role for hGDH2 in primate biology by enabling enzyme recruitment (through an ADP-dependent mechanism dissociated from GTP control) under conditions of high energy utilization (increased conversion of ATP to ADP).

Even though these two changes (Arg443Ser and Gly456Ala) conferred most of the properties of the modern-day hGDH2 enzyme, they were not adequate to fully convert the ancestral enzyme to hGDH2. Indeed, similarly to the Arg443Ser single mutant, the double mutant of hGDH1 (Arg443Ser/Gly456Ala) was found to be essentially inactive (basal activity <1%; little activation by physiologically relevant ADP concentrations), suggesting that additional evolutionary substitutions substantially modified the drastic effect of the Arg443Ser mutation, thus providing the unique properties of hGDH2 [15]. It is of interest in this respect that three additional amino acid changes that occurred in the first step (Ala3Val, Asp142Glu, and Ser174Asn) had a stabilizing effect (by consensus, Figure S3), probably contributing to the enzyme’s functional properties that enabled its survival and subsequent evolution.

The results of our study contribute to the elucidation of the structure–function relationships of hGDH2 through the evolutionary lens. This is important as hGDH2 is involved in human physiology and pathophysiology. Specifically, there is accumulating evidence concerning hGDH2′s putative role in neurodegenerative processes, including Alzheimer’s disease and Parkinson’s disease, and tumorigenesis [24,25,26]. In this respect, we have known that tumors occur in primates in different organs and glands [63]. Also, it has been proven that Alzheimer’s disease is not human-specific but also affects non-human primates [64]. Thus, given its pathophysiological importance for severe human diseases, hGDH2 is becoming an attractive drug target and the study of the structural evolution presented here could assist in rational drug design strategies [39,65,66,67].

### Strengths and Limitations

Of note, AlphaFold Colab predicts GDH structures only at the single subunit level and not for the functional hGDH hexamer. However, the predicted subunit structure includes all the important domains needed for glutamate dehydrogenase function: a glutamate binding region towards the N terminus, a NAD binding domain, and a regulatory domain consisting of the antenna and the pivot helix. It is known that the interactions between different subunits are present in the experimentally determined hexamer, and these interactions influence the structure of each individual subunit. However, when we compared the experimentally determined and the AlphaFold predicted structure of hGDH1 and hGDH2, we did not detect significant deviations. In addition, since in our studies we compared the in silico predicted structures at the individual subunit level during evolution, the hexameric influences are not present in the predicted structures included here. Finally, given that the structural predictions of AlphaFold are similar but not identical to the experimentally determined structures, there is a need for experimental structural data that will verify, complement, and expand in silico AlphaFold-produced data.

## 5. Conclusions

In summary, our most important results from the AlphaFold structure predictions were that (1) GDH2 of modern-day apes is different from hGDH2 and (2) GDH2 in the common ancestor of humans and modern apes (node B in Figure 1) was the steppingstone for the structural and functional evolution of GDH2s in primates. Following this, primate GDHs underwent minor modifications that fine-tuned their enzymatic properties to adapt to the functional needs of modern-day primate nervous and other tissues. These results shed light on the structural/functional relationships of an enzyme that is important for human physiology and disease pathogenesis.

## Figures and Tables

**Table 1:** The AlphaFold predicted local-distance difference test (pLDDT) scores for the proteins studied here.

Protein	AlphaFold pLDDT
Node A (=hGDH1)	93.79
Node B	93.38
Node C	93.80
Node D	93.36
Node E (=hGDH2)	93.85
Chimpanzee (Node E)	93.67
Gorilla (Node D)	93.52
Orangutan (Node C)	93.86
Gibbon (Node B)	93.44

**Table 2:** Qualitative analysis of the predicted effect of amino acid substitutions during evolution. Effect: (D) Destabilizing, (S) Stabilizing. The majority consensus among methods is highlighted in bold (energy trend estimated by three or more methods). Nodes A to E correspond to nodes A to E in Figure 1.

		DynaMut	iSTABLE	PremPS	MaestroWEB	SDM
Human Node A-B	A3V	**S**	D	**S**	**S**	**S**
Human Node A-B	E34K	S	**D**	**D**	**D**	**D**
Human Node A-B	D142E	**S**	**S**	**S**	**S**	**S**
Human Node A-B	S174N	**S**	**S**	D	**S**	**S**
Human Node A-B	R443S	**D**	**D**	**D**	S	**D**
Human Node A-B	G456A	**S**	**S**	D	**S**	**S**
Human Node A-B	N498S	**D**	**D**	**D**	S	**D**
Human Node B-C	V3L	S	**D**	S	**D**	**D**
Human Node B-C	R39Q	S	**D**	**D**	S	**D**
Human Node B-C	K299R	**D**	**D**	**D**	S	S
Human Node B-C	S331T	S	**D**	**D**	**D**	S
Human Node B-C	M370L	**S**	D	**S**	**S**	**S**
Human Node B-C	R470H	S	**D**	**D**	S	**D**
Human Node C-D	I166V	**D**	**D**	**D**	S	S
Human Node C-D	G247R	S	S	**D**	**D**	**D**
Human Node C-D	A321V	D	**S**	**S**	**S**	D
Human Node D-E	R299K	**D**	**D**	S	S	**D**
Human Node D-E	M415L	**D**	**D**	**D**	**D**	S
Chimpanzee Node E	I305L	**D**	**D**	S	**D**	S
Chimpanzee Node E	V321I	S	**D**	**D**	S	**D**
Gorilla Node D	S66C	**D**	**D**	**D**	S	S
Gorilla Node D	K362R	D	**S**	D	**S**	**S**
Gorilla Node D	L365Q	**D**	**D**	**D**	S	S
Gorilla Node D	E439D	**D**	S	**D**	S	**D**
Orangutan Node C	I239N	**D**	**D**	**D**	S	**D**
Orangutan Node C	L240V	**D**	**D**	S	S	**D**
Orangutan Node C	I275V	**D**	**D**	**D**	**D**	**D**
Orangutan Node C	L375V	**D**	**D**	**D**	S	**D**
Orangutan Node C	Q441R	**D**	S	**D**	**D**	**D**
Gibbon Node B	E8K	S	**D**	S	**D**	**D**
Gibbon Node B	T101A	**D**	**D**	**D**	**D**	S
Gibbon Node B	L377V	**D**	**D**	**D**	S	**D**

**Table 3:** Effect of amino acid substitutions on intramolecular interactions. Nodes A to E correspond to nodes A to E in Figure 1.

Evolutionary Step	Amino Acid Substitutions	Bonds Lost	Bonds Gained	Interactions Lost	Interactions Gained
Node A-B	A3V	Ser1		Ser1, Ala5	
Node A-B	E34K	Lys31	Lys31, Asp30	Leu32	
Node A-B	D142E	Gln144, Glu146		Arg178, Gln146, Arg178	Trp182
Node A-B	S174N	Tyr99	Tyr99		Pro137
Node A-B	R443S	Ala447, Phe440, Glu439	Phe440, Ala447		Gln441, Ser445
Node A-B	G456A	His454, Tyr459, Thr460	Val453, His454, Tys459, Ile452		Phe387
Node A-B	N498S		Gly501, Ala500, Phe494	Val496, Phe494, Ile52	
Node B-C	V3L		Ser1		
Node B-C	R39Q				
Node B-C	K299R	Glu296, His302, Gln301, Glu296	Leu295, Glu296,	Phe256	Phe256, Leu295, Ile305
Node B-C	S331T			Lost: Gln334	
Node B-C	M370L	Ile347	Ile347	Ile347, Phe230, Met237	Tyr236, Leu479, Leu481
Node B-C	R470H	Met473, Ala472			
Node C-D	I166V	Pro92	Gly163, Gly160, Ile162		
Node C-D	G247R		Lys249		
Node C-D	A321V	Ile318	Cys323, Lys344		Tyr314, Ile318, Val252, Cys323
Node D-E	R299K	His302, Glu296, Gln301, Asp297		Glu279, Ile305	Gln301, Phe256
Node D-E	M415L	Gln418, His412, Val417		Val417	Leu413

## Data Availability

All datasets analyzed in this work are available from the corresponding author on reasonable request.

## Supplementary Materials

The following supporting information can be downloaded at https://www.mdpi.com/article/10.3390/biom14010022/s1. Figure S1: Superimposed hGDH2 AlphaFold Colab predicted structures during primate evolution; Figure S2: Superposition of the model structure corresponding to the common ape ancestor (blue) with every predicted structure model for each ape (green); Figure S3: Effects of amino acid substitutions occurring during hGDH2 evolution on enzyme stability analyzed by four different webservers; Figure S4: Stabilizing effect of amino acid substitutions occurring during hGDH2 evolution on enzyme stability analyzed by different webservers; Figure S5: Effects of amino acid substitutions occurring during hGDH2 evolution on enzyme stability analyzed by the DynaMut webserver; Table S1: Quantitative analysis of the predicted effect of amino acid substitutions during evolution.
