# Supplementary material for: Structural Evolution of Primate Glutamate Dehydrogenase 2 as Revealed by In Silico Predictions and Experimentally Determined Structures
Source: Biomolecules. 2023 Dec 23;14(1):22. doi: 10.3390/biom14010022 (PMC10812971; doi:10.3390/biom14010022)
Supplement: Supplementary file 1 [file biomolecules-14-00022-s001.zip › biomolecules-2706720-supplementary.pdf]

Supplementary materials

Structural evolution of primate glutamate dehydrogenase 2 as revealed by *in silico* predictions and experimentally determined structures

Ionela Litso[1], Andreas Plaitakis[1], Vasiliki E. Fadouloglou[2], Mary Providaki[3], Michael Kokkinidis[3,4] and Ioannis Zaganas [1,*]

Table S1. Quantitative analysis of the predicted effect of amino acid substitutions during evolution. Effect: (D)Destabilizing, (S) Stabilizing. The majority consensus among methods is highlighted in bold (energy trend estimated by three or more methods).

		**Dynamut**	**iMutant**	**MUpro**	**iSTABLE**	**PremPS**	**MaestroWeb**	**SDM**
	Mutations	ΔΔG (kcal/mol)	ΔΔG (kcal/mol)	Conf. score	Conf. Score	ΔΔG (kcal/mol)	ΔΔGpred (kcal/mol)	ΔΔGpred (kcal/mol)
Human Node A-B	***A3V***	**1.13 (S)**	-0.31(D)	**0.18(S)**	0.63(D)	**-0.17(S)**	**-0.08(S)**	**1.16(S)**
Human Node A-B	***E34K***	0.54 (S)	**-0.81(D)**	**-1.00(D)**	**0.87(D)**	**0.22(D)**	**0.27(D)**	**-0.86(D)**
Human Node A-B	***D142E***	**0.27 (S)**	-0.69(D)	**1.00(S)**	**0.69(S)**	**-0.17(S)**	**-0.44(S)**	**1.25(S)**
Human Node A-B	***S174N***	**0.15 (S)**	**-0.30(S)**	**0.12 (S)**	**0.76(S)**	0.16(D)	**-0.12(S)**	**0.29(S)**
Human Node A-B	***R443S***	**-0.27 (D)**	**-1.11(D)**	**-1.00 (D)**	**0.75(D)**	**0.29(D)**	-0.21(S)	**-0.92(D)**
Human Node A-B	***G456A***	**0.35 (S)**	-0.90(D)	**0.54 (S)**	**0.61(S)**	1.26(D)	**-0.60(S)**	**1.57(S)**
Human Node A-B	***N498S***	**-0.51 (D)**	0.12(S)	**-0.66(D)**	**0.59(D)**	**0.77(D)**	-0.41(S)	**-0.07(D)**
Human Node B-C	***V3L***	0.75(S)	**-0.68(D)**	**-0.28(D)**	**0.87(D)**	-0.22(S)	**0.12(D)**	**-0.15(D)**
Human Node B-C	***R39Q***	0.35(S)	**-0.93(D)**	0.02(S)	**0.53(D)**	**0.34(D)**	-0.13(S)	**-0.06(D)**
Human Node B-C	***K299R***	**-0.51(D)**	**-0.07(D)**	**-0.18(D)**	**0.60(D)**	**0.47(D)**	-0.22(S)	0.01(S)
Human Node B-C	***S331T***	0.12(S)	0.10(S)	**-0.55(D)**	**0.53(D)**	**0.24(D)**	**0.79(D)**	0.23(S)
Human Node B-C	***M370L***	**0.13(S)**	-1.52(D)	-1.00(D)	0.82(D)	**-0.72(S)**	**-0.01(S)**	**0.29(S)**
Human Node B-C	***R470H***	0.08(S)	**-1.34(D)**	**-0.53(D)**	**0.85(D)**	**0.56(D)**	-0.40(S)	**-0.15(D)**
Human Node C-D	***I166V***	**-0.27(D)**	**-0.64(D)**	**-1.00(D)**	**0.81(D)**	**0.26(D)**	-0.08(S)	0.04(S)
Human Node C-D	***G247R***	0.57(S)	-0.22(S)	**-0.40 (D)**	0.56(S)	**0.25(D)**	**0.14(D)**	**-2.36(D)**
Human Node C-D	***A321V***	-0.06(D)	**-0.02(S)**	**0.02(S)**	**0.69(S)**	**-0.07(S)**	**-0.19(S)**	-0.47(D)
Human Node D-E	***R299K***	**-0.83(D)**	**-0.89(D)**	**-1.00 (D)**	**0.67(D)**	-0.61(S)	-0.08(S)	**-0.72(D)**
Human Node D-E	***M415L***	**-0.02(D)**	**-0.95(D)**	**-0.60 (D)**	**0.70(D)**	**0.48(D)**	**0.14(D)**	0.54(S)
Chimpanzee Node E	***I305L***	**-0.49(D)**	**-0.50 (D)**	**-0.04 (D)**	**0.61(D)**	-0.183(S)	**0.09(D)**	0.09(S)
Chimpanzee Node E	***V321I***	0.15(S)	**-0.25(D)**	**-1.00 (D)**	**0.78(D)**	**0.099(D)**	-0.05(S)	**-0.39(D)**
Gorilla Node D	***S66C***	**-0.03(D)**	**-1.06(D)**	**-0.72 (D)**	**0.62(D)**	**0.44(D)**	-0.37(S)	0.7(S)
Gorilla Node D	***K362R***	-0.12 (D)	-0.36(D)	**0.39 (S)**	**0.58(S)**	0.21(D)	**-0.35(S)**	**0.26(S)**
Gorilla Node D	***L365Q***	**-0.50(D)**	**-2.38(D)**	**-1.00(D)**	**0.85(D)**	**1.64(D)**	-0.52(S)	0.17(S)
Gorilla Node D	***E439D***	**-0.53(D)**	-0.28(S)	**-0.98(D)**	0.58(S)	**0.06(D)**	-0.08(S)	**-1.48(D)**
Orangutan Node C	***I239N***	**-0.38(D)**	**-1.71(D)**	**-1.00(D)**	**0.81(D)**	**0.67(D)**	-0.77(S)	**-0.86(D)**
Orangutan Node C	***L240V***	**-1.53(D)**	**-1.51(D)**	**-0.46(D)**	**0.88(D)**	-0.28(S)	-0.27(S)	**-3.06(D)**
Orangutan Node C	***I275V***	**-1.02(D)**	**-0.93(D)**	**-1.00(D)**	**0.83(D)**	**0.51(D)**	**0.06(D)**	**-1.91(D)**
Orangutan Node C	***L375V***	**-1.57(D)**	**-1.75(D)**	**-1.00(D)**	**0.80(D)**	**1.14(D)**	-0.35(S)	**-3.07(D)**
Orangutan Node C	***Q441R***	**-0.50(D)**	0.19(S)	**-0.76(D)**	0.57(S)	**0.3(D)**	**0.01(D)**	**-0.23(D)**
Gibbon Node B	***E8K***	0.35(S)	**-1.00(D)**	**-1.00(D)**	**0.76(D)**	-0.19(S)	**0.35(D)**	**-0.16(D)**
Gibbon Node B	***T101A***	**-0.54(D)**	**-1.16(D)**	**-0.77(D)**	**0.71(D)**	**0.36(D)**	**0.16(D)**	0.12(S)
Gibbon Node B	***L377V***	**-1.15(D)**	**-1.70(D)**	**-1.00(D)**	**0.81(D)**	**0.79(D)**	-0.27(S)	**-2.90(D)**

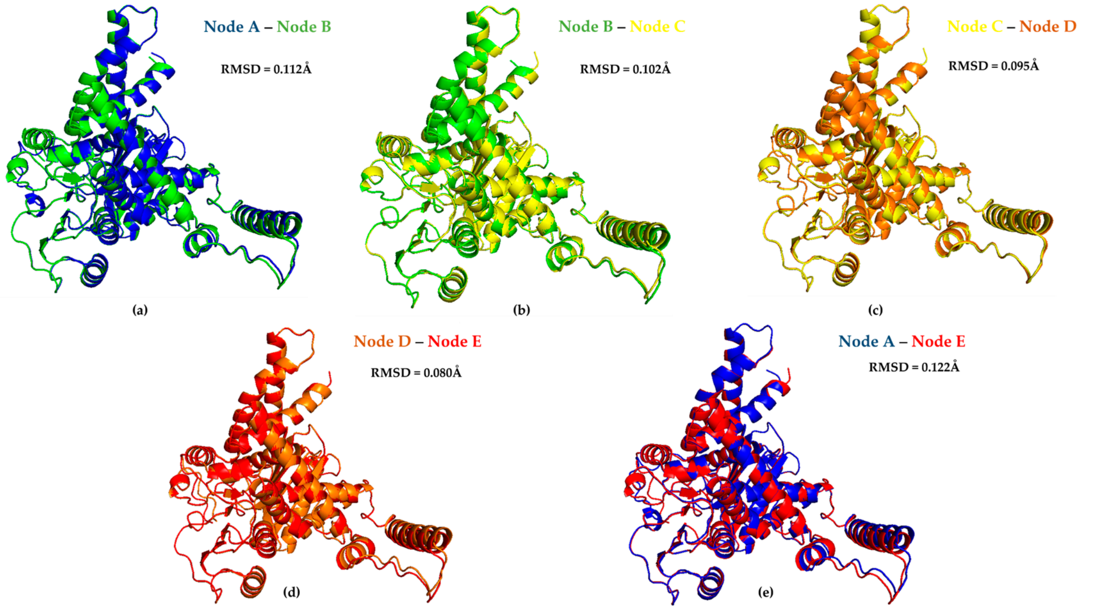

Figure S1. Superimposed hGDH2 AlphaFold Colab predicted structures during primate evolution (see Figure 1 for Node designation). (a) NodeA (blue) -NodeB (green). The RMSD value was estimated to be 0.112Å. (b) NodeB (green) – NodeC (yellow). RMSD value:0.102Å. (c) NodeC (yellow) – NodeD (orange). RMSD value: 0.095Å. (d) NodeD (orange) – NodeE (red),RMSD value: 0.080Å. (e) Node A (blue) -Node E (red), RMSD value: 0.122Å. Nodes A to E correspond to nodes A to E in Figure 1.

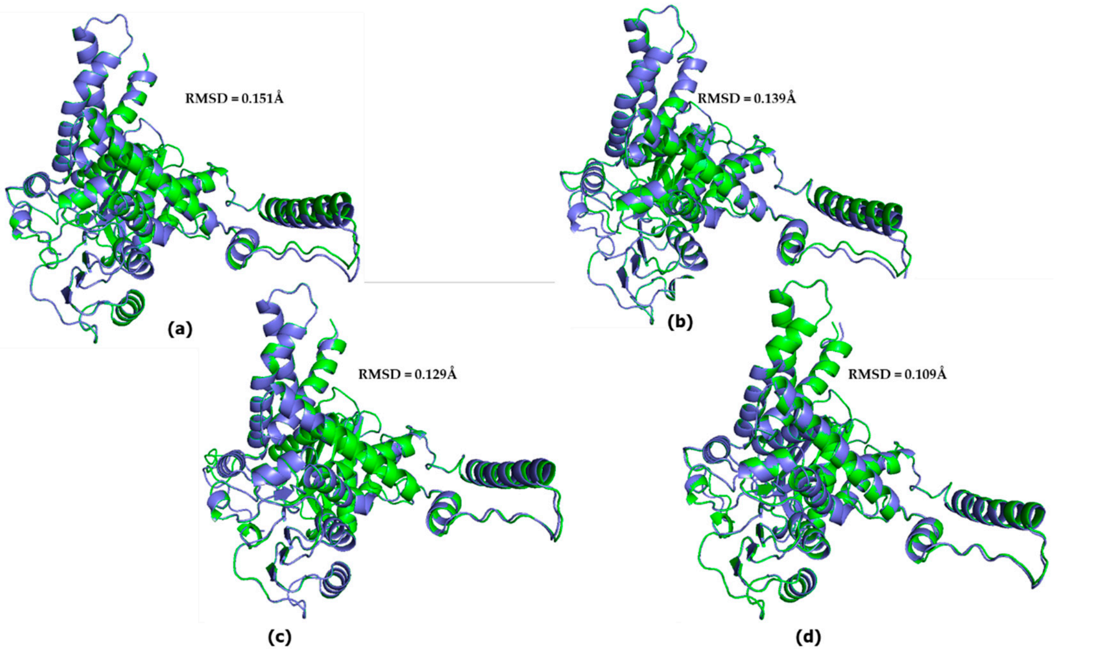

Figure S2. Superposition of the model structure corresponding to the common ape ancestor (blue) with every predicted structure model for each ape (green). (a) chimpanzee GDH2-common ape ancestor. The RMSD value was estimated to be 0.151Å. (b) gorilla-common ape ancestor. The RMSD value was estimated to be 0.139Å. (c) orangutan-common ape ancestor. The RMSD value was estimated to be 0.129Å. (d) Gibbon-common ape ancestor. The RMSD value was estimated to be 0.109Å.

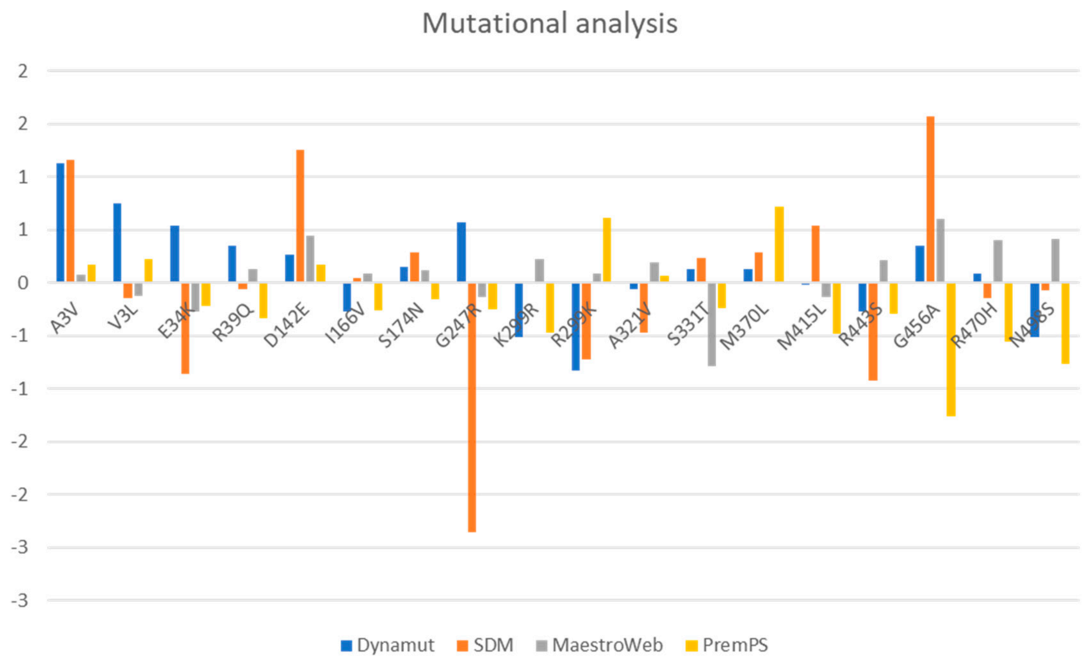

Figure S3. Effects of amino acid substitutions occurring during hGDH2 evolution on enzyme stability analyzed by four different webservers (Dynamut, SDM, Maestroweb, PremPS). Results from MastroWeb and PremPS have been multiples by -1 so that all values indicating stabilization are above zero and all values indicating destabilization are below zero.

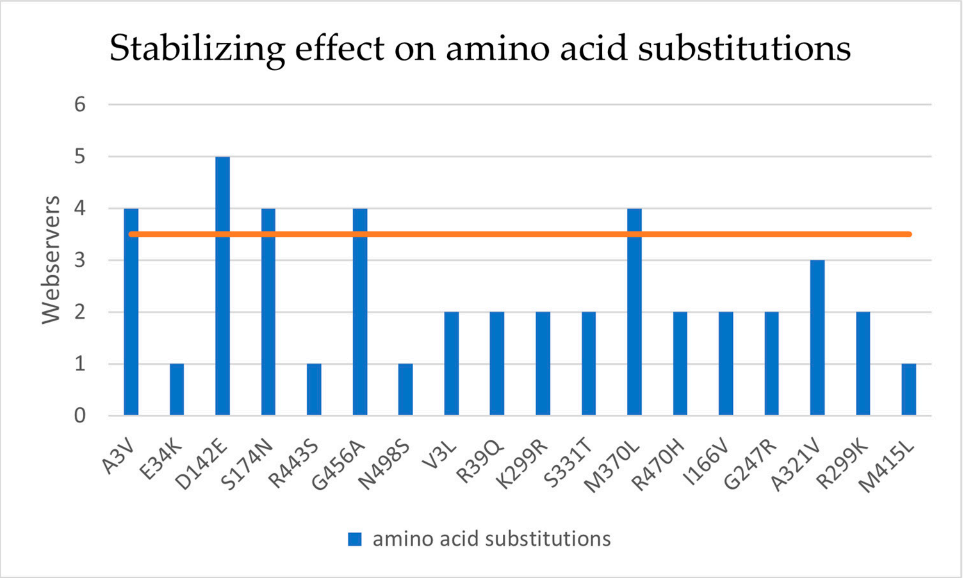

Figure S4. Stabilizing effect of amino acid substitutions occurring during hGDH2 evolution on enzyme stability analyzed by the different webservers (PremPS, iMutant, DynaMut, MAESTRO, MUpro, iSTABLE, SDM). Bars above the orange line indicate that the mutation has a stabilizing effect by consensus (more than 3 servers predict stabilization).

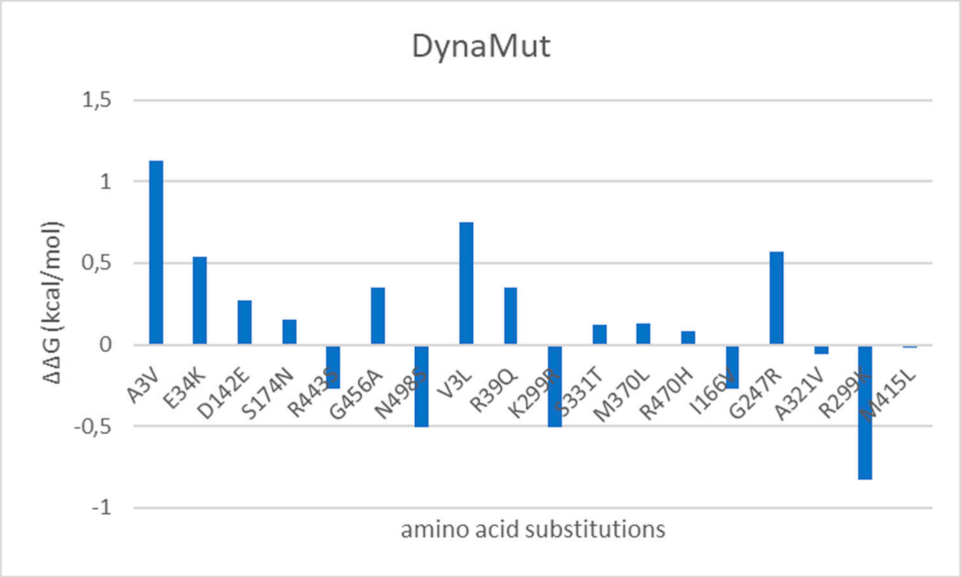

Figure S5. Effects of amino acid substitutions occurring during hGDH2 evolution on enzyme stability analyzed by the DynaMut webserver. ΔΔG > 0 corresponds to stabilizing effect whereas ΔΔG < 0 to destabilizing effect.
